# Association between childhood trauma, intimate partner violence, and perceived parental competence among women abusing amphetamine-type stimulant

**DOI:** 10.3389/fpsyt.2022.994324

**Published:** 2023-01-04

**Authors:** Suzaily Wahab, Rubini Sivarajah, Amirul Danial Azmi, Norliza Chemi, Raynuha Mahadevan

**Affiliations:** ^1^Department of Psychiatry, Universiti Kebangsaan Malaysia Medical Centre, Bandar Tun Razak, Malaysia; ^2^Department of Psychiatry, Hospital Kajang, Bandar Kajang, Selangor, Malaysia

**Keywords:** child abuse, amphetamine type stimulant (ATS), domestic violence, women's health, parental competency

## Abstract

**Introduction:**

This cross-sectional study examines the correlation between childhood trauma, intimate partner violence (IPV), and parenting self-efficacy among women who reported using amphetamine-type stimulants (ATS) in an institutional drug rehabilitation center.

**Methods:**

A total of 106 participants were recruited by purposive sampling, of which 88 were mothers. Questionnaires were used to collect sociodemographic data and study variables.

**Results:**

Most of these women had experienced emotional abuse, sexual abuse, and physical neglect in their childhood. IPV assessments revealed that 70.5% (*n* = 74) and 30.5% (*n* = 32) had experienced physical and sexual violence, respectively. In terms of parenting competency, they scored 79.5% for self-efficacy and 54.4% for parenting satisfaction. Childhood emotional abuse significantly increases the odds of individuals experiencing sexual violence by 20.9%.

**Discussion:**

We found that childhood trauma and IPV did not have a significant relationship with parenting efficacy. Conversely, childhood emotional abuse and physical abuse were negatively correlated to parenting satisfaction. It is imperative that any form of childhood abuse be recognized and stopped early to reduce the harm it brings to women later in life.

## Introduction

There is considerable concern about the increase in substance use globally. It was reported that about 269 million people worldwide used recreational drugs in 2018, a 30% increase from 2009. According to the latest World Drug Report, more than 35 million people have been affected by drug use disorders (1). In the Malaysian context, the rise of substance use and the associated manifestation with criminality, particularly among women, has reached a concerning level. The National Anti-Drug Agency (NADA) documented that the total number of people who used drugs (PWUD) caught was 20,643 in the year 2020, with 95.3% men and 4.7% women. Although the number of women involved in drug abuse is smaller than that of men, there has been a considerable increase from 580 women per case in 2010 to 974 women per case in 2020, which was an almost 2-fold increase within the past decade (2, 3).

In Malaysia, the predominant substance used was amphetamine-type stimulants (ATS), with 65.2% of PWUD caught using ATS, compared with 30.9% of opiates and 2.6% of cannabis in 2020 (2). Kelantan et al. states were reported to have the highest involvement in drug abuse when compared with other states in Malaysia (4). There also appeared to be a difference in addiction trends among women with regard to the type of substance used. Women in Malaysia were reported to use methamphetamine (MA) and heroin compared with other countries in America and Europe, where pharmaceutical prescription drugs are the most commonly used drugs (5). MA is also the most used illicit substance currently, and it is widely used by vulnerable populations of women around the world. These include sex workers, homeless women, psychiatric patients, sexual minorities, and pregnant women. MA is a synthetic psychostimulant with a high risk of developing addiction. Its stimulant effects include increased wakefulness, focus, and euphoria. However, with chronic use, the side effects manifested from this drug are debilitating, including depression, psychotic episodes, paranoia, aggression, and violence. Women with MA use disorder are at a significantly higher risk of perpetrating and being victims of violence. These effects are particularly worrying as MA use affects pregnant mothers, specifically causing small for gestational age and low birth weight (6).

Various studies have explored the risk factors that lead to substance use. Childhood trauma and violent victimization were reported as the most common risk factors. Childhood trauma refers to various types of maltreatment experienced by children younger than 18 years (7). Child maltreatment refers to physical and emotional mistreatment, sexual abuse, neglect, negligent treatment of children, and their commercial or other forms of exploitation (8). These maltreatments are suggested to trigger the use of the substance as a coping strategy to alleviate the pain of being maltreated or indirectly by causing a criminal lifestyle that mediates contact with substance use (8). A local study by Wahab et al. (9) reported that peer and sibling victimization was positively correlated with lifetime use of illicit substances. It was also reported that young women with a history of childhood sexual abuse were more likely to have psychiatric comorbidities, such as depression (10). Moreover, childhood trauma and psychiatric comorbidities were also reported among women who use MA (6).

Intimate partner violence (IPV) is a pattern of violence, abuse, or intimidation used to control or maintain power over a partner in an intimate relationship. An intimate partner is described as a romantic or sexual partner and includes spouses, boyfriends, girlfriends, and people with whom they dated, were seeing, or “hooked up” (11). IPV varies in frequency and severity, ranging from one episode that might or might not have a lasting impact to chronic and severe episodes for years. Various forms of IPV include physical, emotional, psychological, sexual, social, and financial abuse. The following findings were reported in the study by Smith et al. (12):

- About one in four women experienced contact sexual violence, physical violence, and/or stalking by an intimate partner and reported an IPV-related impact during their lifetime.
- Regarding specific subtypes of IPV, about 18.3% of women experienced contact sexual violence, 30.6% experienced physical violence (21.4% experienced severe physical violence), and 10.4% experienced stalking during their lifetime.
- An estimated 71%, or nearly 31.0 million, reported being victims of contact sexual violence, physical violence, and/or stalking by an intimate partner. They first experienced these or other forms of violence by that partner before the age of 25 years, and one in four female victims (25.8% or about 11.3 million victims) first experienced IPV before the age of 18 years.
- In Malaysia, the prevalence of IPV ranges between 4.9 and 35.9%, with psychological and emotional abuse being the most prevalent form of IPV (13).

There is ample evidence to suggest a close relationship between IPV and substance use. IPV is commonly reported by female MA users and is considered a warning sign of more severe forms of violence by male users (6). The history of sexual abuse of women in substance use treatment ranges from 30 to 75%, leading many to suggest sexual trauma as a key contributor to women's drug abuse (14). Studies have shown that MA use is closely associated with various forms of violence and aggression, including IPV (15). A similar trend was reported in a local study where women abusing drugs and having a history of childhood trauma were significantly associated with experiencing emotional and physical violence during pregnancy (16). Over the last decade, researchers have examined the mechanisms by which childhood abuse can lead to adulthood IPV (17). According to the social learning theory, children raised in a violent home will see aggression as an effective response to conflict because of observational learning, modeling, and direct behavioral conditioning. As a result, children who are exposed to violence in their early life are more likely to re-experience or replicate violence in their adult interpersonal relationships (18). Childhood trauma is also associated with an earlier onset of initiating MA. Emotional abuse and physical abuse are reported to be predictive factors to the earlier age of MA initiation among dependents (19). The impact of childhood trauma is correlated not only with the age of onset but also with the severity of dependence (20).

Pregnancy facilitates a life change in which women begin to plan for their child's birth and cultivate a maternal function, but parenting self-efficacy factors for women with substance use have been studied infrequently. Previous studies have shown that higher levels of social support and family empowerment were related to increased parenting self-efficacy among mothers using illicit substances, especially during their vulnerable period. Studies have shown that social networks and family support have reduced stress, allowing a more fluid shift to motherhood (21). A previous study has shown duality in the experience of motherhood among some women using substances. On the one hand, there were some idealized views of motherhood, and some built a more adapted version of the good mother model to fit their reality. This model refers to what we call “the deviant good mother” model (22). In the idealized view of motherhood, women consider motherhood to fulfill their emotional needs or heal those wounds, mainly in their relationships with their mothers. Some women with drug use disorders confess to repeating the unhealthy parenting patterns they witnessed during their childhood, but they are willing to improve their parenting methods now. Changing these habits and raising their children in a different way can help them see themselves as distinct from their parents, as doing what is necessary for their children, and thus redeeming parts of their maternal identity (22).

On the other hand, some seemed to adjust their conception of motherhood so as not to contradict this lifestyle. These women expressed their vision of motherhood and tried to prove that they could be “good mothers” just like others. These mothers may be trying to justify themselves or compensate for their deviant behavior.

In Malaysia, the number of drug treatment and rehabilitation program centers for women is limited compared with that for men. According to the World Drug Survey, this is due to a lack of evidence-based treatment and recovery services for women. Most substance-related studies have excluded women because it is believed that women are biologically more complex than men and that women are too preoccupied with caring for their children to engage in studies. However, it is important to remember that women face unique issues regarding substance use with regard to pregnancy and child-rearing capacity. As the predominant caregiver, women play an important role in parenting children. Thus, violent behaviors resulting from MA use may be detrimental to the ability to carry out parental roles and may have an everlasting effect on their children. Thus, understanding a woman's needs concerning past, childhood, sexual, and other types of violence; mental illness; and parenting is critical.

This study aims to investigate the association between childhood trauma and IPV and perceived parent self-efficacy among women with amphetamine-type stimulants (ATS) use disorder. This study has clinical value in identifying and examining associations with amphetamine-type stimulants and allows for further research on ways to mitigate these effects on drug usage.

## Method

### Study design and setting

This cross-sectional study was conducted from December 2020 to March 2021 on consenting women with substance use disorder undergoing rehabilitation in a substance rehabilitation center. This rehabilitation center was chosen because it is the only center accepting women with substance use disorders. Data were gathered through a video conferencing platform. Women in this rehabilitation center came from other states, such as Johor and Penang, but the majority of them was from the East Coast. There are two ways to be admitted to the center: by a court order or on a voluntary basis, and they are expected to stay for a maximum of 2 years. Upon completion of their rehabilitation, the participants are allowed to return to the community and will undergo surveillance by their respective district NADA. During their stay at the rehabilitation center, the participants were not allowed to access any type of substance whatsoever. Their children were not permitted to accompany them to the rehabilitation center. However, if they give birth during their stay, they are allowed to have their child with them. Clients can communicate with their children and spouses through letters, phone calls, and family visits.

### Data collection

The study population consisted of women enrolled in a rehabilitation center during this period. The study employed purposive sampling in which all the clients in the rehabilitation center were screened using the inclusion and exclusion criteria. This method was employed to have a larger sample size and more reliable results. The inclusion criteria were all women aged between 18 and 65 years, all those who were able to communicate and understand in either Bahasa Malaysia or English, women with a history of ATS use and able to read and write fluently in Bahasa Malaysia, and all those who were able to give informed and written consent. The clients who met these criteria were then informed and invited to participate in this study to which they could participate and withdraw at any time during the study. The participation in the study was on a voluntary basis.

Given the current social distancing practice, data were collected using online interview sessions and Google Forms. The participants were interviewed through an online video conference tool using the rehabilitation center facilities and in a private room to ensure their privacy. The researcher explained the study information and the objectives during the interview conducted in an online format. The online form included the study information, objectives, inclusion and exclusion criteria, and a consent form to which the clients would have to click on “yes” before being able to proceed with the self-rated questionnaires. Each individual was given a set of five questionnaires, whereby three of them were self-reported, and the other two were interviewer-rated. The participants used Google Forms to complete self-reported questionnaires, which were (1) social demographics, (2) Childhood Trauma Questionnaire Short Form (CTQ-SF), and (3) Parenting Sense of Competence Scale (PSOC). The interviewer-rated questionnaires used were the Mini International Neuropsychiatric Interview (MINI) 6.0 Malay version, and the Women's Health and Life Experiences Questionnaire was given to the participants in an online interview. Each participant was provided 30–45 min to answer a set of questionnaires. Confidentiality was guaranteed for all participants. The participants were encouraged to seek assistance while answering the self-reported questionnaires. An officer was briefed regarding the questionnaire to assist these women. This study obtained ethical approval from the Medical Ethics Committee of the institution and National Medical Research Register.

### Study instruments

Before this study began, permission from the original authors for the questionnaires MINI 6.0, CTQ-SF, Women's Health and Life Experiences Questionnaire, and PSOC was obtained.

#### Patient clinical-demographic questionnaire

This questionnaire collects information from each participant about age, ethnicity, religion, marital status, level of education, previous employment status, whether they had children, household income, and health-related variables.

#### MINI 6.0 Malay version

MINI 6.0 was only used to diagnose participants with substance use disorder (SUD). The latest English version of MINI 7.2, designed as a brief structured interview on the major psychiatric disorders in DSM-5 to diagnose SUD, requires two or more questions from the J2 section to be coded as yes. The MINI 6.0 Malay version was shown to have a reliable and valid psychometric property (23). This is a brief structured interview on the major psychiatric disorders in DSM-IV. For a diagnosis of current substance dependence, it is needed to have three or more answers in section J2 coding yes. This study adopted the MINI 6.0 Malay version because it is more suitable for the population setting (24). However, the authors also ensured that the participants fulfilled the DSM 5 criteria for substance use disorder.

#### CTQ-SF

The instrument was designed to include 25 items (25) to measure traumatic experiences during childhood. The instrument contains 25 items divided into the following five domains: physical, emotional, and sexual abuse, and physical and emotional neglect domain. An additional three-item minimization/denial scale was included to measure any underreporting of maltreatment. This instrument uses a five-point Likert scale to measure the response with the following scales: 1 = never, 2 = rarely, 3 = sometimes, 4 = often, and 5 = very often. The sum of the item score for each scale is used to compute the clinical score, with higher scores indicating a more severe experience of childhood maltreatment. This study attempts to translate the questionnaire into a Bahasa Malaysia version. Forward and expert panel translation was done by a language and healthcare expert. The translated questionnaire was pretested among 30 healthcare staff in the institute medical center to ensure that the wording of the questions was correct and that they understood the questionnaire. The internal consistency of this translated CTQ was 0.62.

#### Women's health and life experiences questionnaire Malay version

The Women's Health and Life Experiences Questionnaire was obtained from the WHO multi-country study on women's health and domestic violence against women. This questionnaire measures domestic violence prevalence, health implications, and risk factors. It consists of 20 specific items that measure four types of IPV act, such as controlling behaviors, emotional violence, physical violence, and sexual violence (26). Section 7 of this questionnaire measures physical and sexual violence. Answering yes to any of the questions on physical and sexual violence corresponds to having experienced physical and/or sexual violence. The Malay version of the WHO Women's Health and Life Experiences Questionnaire is a valid and reliable measure of women's health and experiences of IPV in Malaysia with a Cronbach's alpha value of 0.767–0.858 for all domains (27).

#### PSOC

The PSOC is a valid and reliable tool developed by Gibaud–Wallston and Wandersman (28). The PSOC is a 17-item scale with two domains measuring parenting self-esteem. Each item is rated on a six-point Likert scale ranging from strongly agree to strongly disagree, with nine items falling under the satisfaction domain and seven items under the efficacy domain. The satisfaction domain examines mothers' anxiety, motivation, and frustration, while the efficacy domain measures mothers' perception of competence, capability levels, and problem-solving abilities in their parental role (29, 30). This study attempts to translate the questionnaire into a Bahasa Malaysia version. Forward and expert panel translation was done by a language and healthcare expert. The translated questionnaire underwent expert testing on 30 healthcare staff in the institute medical center. All participants understood the translated parenting sense of competence scale, and the internal consistency was 0.6.

### Statistical analysis

In the descriptive analysis, mean with standard deviation or median with interquartile was used to present continuous data, depending on the normality. In addition, the frequency with percentage was used to present categorical variables. For inferential analysis, an independent *t*-test, Mann–Whitney U test, chi-square test, Pearson correlation, or Spearman ranked correlation was used to determine significant differences between childhood trauma and IPV and parenting competency among women in a rehabilitation center. Considering regression, a simple logistic was used to determine the association between independent variables with binary outcomes. On the other hand, linear regression was used to check the relationship between all independent variables and continuous outcome variables (31). In this study, the independent variable is childhood trauma, the mediating variable is IPV, and the outcome is parenting competency. These statistical tests were performed on International Business Machines Corporation's Statistical Package for the Social Sciences (SPSS) version 22.

## Results

### Respondents' sociodemographic characteristics

There were 128 women in the rehabilitation center, of which 22 were not included in this study as they did not meet the inclusion and exclusion criteria (two of them were younger than 18 years, and 20 still had some withdrawal symptoms). The normality test results showed that age, childhood trauma (minimization/denial), and parenting sense of competence scale (both parental efficacy and satisfaction domains) were normally distributed. Hence, a mean with standard deviation was used to present those variables, whereas the others were presented as median with interquartile range. In Table 1, the mean age of the 106 participants was 33.5 (SD = 7.13) years. A total of 92.5% (*n* = 98) of the participants were Malay, and 46.2% (*n* = 49) of them were widowed, single mothers, divorced, or in the process of divorcing, and about 83% (*n* = 88) of them had children. Regarding education and employment, 87.7% (*n* = 93) completed secondary education, and 71.7% (*n* = 76) had a full-time job. Most participants resided in the city with a monthly household income of below RM 2,000. Regarding the health-related profile, 25.5% (*n* = 27) of them had a history of general illness. All participants were diagnosed with substance use disorder, 10% (*n* = 11) were diagnosed with anxiety, depression, or psychosis/schizophrenia, and 8.5% (*n* = 9) of these women had received psychiatry treatment before. About 11% (*n* = 12) of them had an episode of attempted suicide. About half, 48.1% (*n* = 51), of the participants had family members who used substances. In addition, 53.8% (*n* = 57) of the participants had friends who used substances, followed by their spouse at 37.7% (*n* = 40) and others at 8.5% (*n* = 9).

**Table 1:** Sociodemographic characteristics, health profiles, childhood trauma, intimate partner violence, and types of substance used variables among women with substance use in a rehabilitation center (*n* = 106).

**Variables**	**Median (IQR)/ Mean (SD)**	***n*** **(%)**
**Age**	33.5 (7.13)^1^	
**Ethnicity**		
Malay		98 (92.5)
Chinese		1 (0.9)
Indian		1 (0.9)
Others		6 (5.7)
**Marital status**		
Widowed/Single		49 (46.2)
mum/Divorced/Process of divorcing		21 (19.8)
Single		36 (34.0)
Married		
**Children**		
No		18 (17.0)
Yes		88 (83.0)
**Education level**		
Diploma/Degree		8 (7.5)
Secondary		93 (87.7)
Primary		5 (4.7)
**Job**		
Jobless/others		26 (24.5)
Part-time		4 (3.8)
Full-time		76 (71.7)
**Stay**		
City		77 (72.6)
Outside city		29 (27.4)
**Household income (monthly)**		
<RM2,000		74 (69.8)
RM2,001–RM5,000		27 (25.5)
RM5,001–RM10,000		4 (3.8)
>RM10,000		1 (0.9)
**History with general illness**		
No		79 (74.5)
Yes		27 (25.5)
**History of mental illness**		
Anxiety		1 (0.9)
Depression		9 (8.5)
Psychosis/Schizophrenia		1 (0.9)
No mental illness		95 (89.6)
**Psychiatry treatment**		
No		97 (91.5)
Yes		9 (8.5)
**History of attempted suicide**		
No		94 (88.7)
Yes		12 (11.3)
**History of family members who used substances**		
No		55 (51.9)
Yes		51 (48.1)
**Known persons who used substances**		
Spouse		40 (37.7)
Friends		57 (53.8)
Others		9 (8.5)
**Childhood trauma**		
Emotional abuse	11.0 (5.0)^b^	
Physical abuse	9.0 (2.0)^b^	
Sexual abuse	13.0 (0.0)^b^	
Emotional neglect	6.0 (5.0)^b^	
Physical neglect	14.0 (3.0)^b^	
Minimization/Denial	1.5 (0.99)^a^	
**Physical violence (*****n*** **=** **105)**		
No		31 (29.5)
Yes		74 (70.5)
**Sexual violence (*****n*** **=** **105)**		
No		73 (69.5)
Yes		32 (30.5)
**Mostly used substance**		
Hallucinogen		1 (0.9)
Cocaine		1 (0.9)
Narcotic		12 (11.3)
Stimulant (Including Amphetamine)		92 (86.8)
**Substance used in the past 12 months**		
Stimulants		104 (98.1)
Other substances		2 (1.9)
**Substance dependency (current)**		
No		0
Yes		106 (100.0)

IQR, interquartile range; SD, standard deviation.

a = mean (SD);

b = median (IQR).

#### Respondents' history of violence

Regarding childhood trauma, emotional abuse, sexual abuse, and physical neglect were most frequently answered. During adulthood, 70.5% (*n* = 74) of the respondents experienced physical violence by their intimate partner, and conversely, 30.5% (*n* = 32) of them encountered sexual violence from their partner.

#### Respondents' drug use history

All of the study participants used a type of stimulant before. A series of substance-related items in the MINI 6.0 Malay version instrument indicated that more than 90% of the participants fulfilled the criteria for substance use, ultimately resulting in all individuals being clinically diagnosed with current substance dependency or use disorder.

### Association between childhood trauma and IPV

Tables 2, 3 illustrate the relationship between childhood trauma domains and IPV (physical and sexual). A Mann–Whitney U test showed that individuals with sexual violence tend to have a higher median score in emotional abuse during childhood than the participants without experiencing sexual violence. The median score showed significance at a 0.05 level. A simple logistic regression showed that every increment of one unit of emotional abuse score significantly increases the odds of individuals experiencing sexual violence by 20.9% (95% CI: 1.044, 1.401). Also, an independent test deduced that women who suffered from physical violence had a significantly lower minimization/denial score than their counterparts (*p* = 0.04). However, a simple logistic regression showed no significant association between minimization and denial score with physical violence in adulthood, as the *p*-value was more than 0.05 (0.055). Other childhood trauma domains, such as physical abuse, sexual abuse, emotional neglect, and physical neglect, were not significantly associated with intimate partner physical and sexual violence.

**Table 2:** Association between childhood trauma and intimate partner violence (*n* = 105).

**Childhood trauma**	**Intimate partner violence** **(physical violence)**		**U /t statistic** ***p***	**Intimate partner violence** **(sexual violence)**		**U-statistic** ***p***
	**No**	**Yes**		**No**	**Yes**	
Emotional abuse Median (IQR)	11.0 (4.0)	12.0 (5.0)	960.000^a^ 0.178	11.0 (4.0)	12.5 (4.0)	812.000^a^ 0.011^*^
Physical abuse Median (IQR)	9.0 (2.0)	9.0 (2.0)	1084.000^a^ 0.637	9.0 (2.0)	10.0 (2.0)	1068.500^a^ 0.461
Sexual abuse Median (IQR)	13.0 (0.0)	13.0 (0.0)	1062.500^a^ 0.466	13.0 (0.0)	13.0 (1.0)	1163.000^a^ 0.966
Emotional neglect Median (IQR)	6.0 (2.0)	6.0 (6.0)	970.000^a^ 0.195	6.0 (4.0)	7.0 (7.0)	1015.500^a^ 0.269
Physical neglect Median (IQR)	13.0 (3.0)	14.0 (3.0)	977.000^a^ 0.211	13.0 (3.0)	14.0 (3.0)	1045.000^a^ 0.370
Minimization/Denial Mean (SD)	1.8 (0.86)	1.4 (1.01)	2.095^b^ 0.040^*^	1.6 (0.97)	1.5 (1.02)	0.361^b^ 0.719

U, Mann–Whitney U-test; t, independent t-test; IQR, interquartile range; SD, standard deviation;

a = U statistic;

b = t-statistic.

* = p-value significance at the 0.05 level.

**Table 3:** Simple logistic regression on childhood trauma subdomains in association with physical and sexual violence.

**Childhood trauma**	**Physical violence (yes)**		**Sexual violence (yes)**	
	**OR (95% CI)**	***p***	**OR (95% CI)**	***p***
Emotional abuse	1.085 (0.929, 1.267)	0.305	1.209 (1.044, 1.401)	0.011^*^
Physical abuse	1.052 (0.895, 1.237)	0.540	1.037 (0.898, 1.197)	0.625
Sexual abuse	1.045 (0.826, 1.321)	0.715	0.874 (0.682, 1.120)	0.288
Emotional neglect	1.096 (0.972, 1.236)	0.134	1.055 (0.956, 1.163)	0.287
Physical neglect	1.138 (0.902, 1.436)	0.275	1.020 (0.823, 1.265)	0.853
Minimization/Denial	0.639 (0.405, 1.010)	0.055	0.924 (0.604, 1.414)	0.716

OR, odds ratio; CI, confidence interval;

* *p*-value significance at the 0.05 level.

### Respondents' parenting sense of competency

We also investigated the parenting sense of competency from 87 (1 with missing data) participants with children, as shown in Table 1. The mean of the participants' perceived parenting efficacy was 33.3 (0.95, CI 31.7–34.9), and the mean score for parental satisfaction was 29.1 (SD = 5.14). In the parental satisfaction domain, five items, namely, items 3, 5, 8, 9, and 14, had an unexpectedly high proportion (62.5–88.7%) of the participants who agreed to those negative statements. Item 3 focuses on individual accomplishment, item 5 looks into self-perception on being a good mother, and item 8 assesses the difficulty in being a parent by not knowing whether their actions are good or bad. The remaining two items were items 9 and 14, focusing on valuing self as a worthless mother and motivation to be a good mother.

#### Correlation between PSOC with childhood trauma and IPV

Tables 4, 5 show the relationship between the PSOC score with childhood trauma domains and IPV, respectively. The correlation analysis in Table 4 shows that emotional neglect was negatively correlated with parents' perceived efficacy (ρ: −0.308, *p* < 0.01). On the other hand, emotional abuse (ρ: −0.286, *p* < 0.01) and physical abuse (ρ: −0.267, *p* < 0.05) during childhood were both negatively correlated with satisfaction. Among the 87 individuals with IPV, mothers who had encountered sexual violence scored the highest for perceived parenting efficacy (mean = 35.0, SD = 6.31) but lowest for the parenting satisfaction score (mean = 28.5, SD = 4.84). However, the independent *t*-test showed no statistical mean difference in parenting efficacy and satisfaction scale between those with or without IPV, as shown in Table 5.

**Table 4:** Correlation between childhood trauma with parenting competence scale domains (efficacy and satisfaction).

**Childhood trauma**	**Efficacy**	**Satisfaction**
Emotional abuse^b^	−0.031	–0.286^**^
Physical abuse^b^	−0.030	–0.267^*^
Sexual abuse^b^	−0.146	−0.021
Emotional neglect^b^	−0.308^**^	−0.177
Physical neglect^b^	0.042	−0.049
Minimization/Denial^a^	0.139	0.015

a Pearson correlation coefficient;

b Spearman ranked correlation coefficient;

* *p* < 0.05;

** *p* < 0.01.

**Table 5:** Mean difference parenting efficacy and satisfaction stratified by physical and sexual violence.

**Intimate partner violence**	**Efficacy**	**Satisfaction**
	**mean (SD)**	**mean (SD)**
**Physical**		
No	33.8 (7.51)	29.0 (5.00)
Yes	33.1 (7.28)	29.1 (5.23)
**Sexual**		
No	32.5 (7.65)	29.3 (5.29)
Yes	35.0 (6.31)	28.5 (4.84)

SD, standard deviation; *p < 0.05.

Multiple and simple linear regression, as shown in Table 6, showed that both childhood trauma and IPV did not have a significant relationship with the efficacy scale. Conversely, two childhood trauma domains were related to parental satisfaction, notably emotional abuse and physical abuse. For every unit increase in emotional abuse, parental satisfaction decreases by 0.482 unit (95% CI: −0.859, −0.105, *p* = 0.013). In the physical abuse domain, every unit increment significantly reduced the parental satisfaction score by 0.518 unit (95% CI: −0.870, −0.166, *p* = 0.004). After accounting for other domains in the childhood trauma questionnaire and IPV, the only factor that remained significantly associated with parenting satisfaction was physical abuse (adjusted B: −0.439; 95% CI: −0.863, −0.014; *p* = 0.043). Emotional abuse was no longer associated with parenting satisfaction following the adjustment (adjusted B: −0.225; 95% CI: −0.769, 0.320; *p* = 0.414).

**Table 6:** Simple and multiple linear regression analyses on relationship between childhood trauma domains and intimate partner violence with parental satisfaction.

**Risk factors**	**Satisfaction**		**Satisfaction**	
	**B (95% CI)**	***p***	**Adjusted B (95%CI)**	***p***
**Childhood trauma**			
Emotional abuse	−0.482 (−0.859, −0.105)	0.013^*^	−0.225 (−0.769,0.320)	0.414
Physical abuse	−0.518 (−0.870, −0.166)	0.004^**^	−0.439 (−0.863, −0.014)	0.043^*^
Sexual abuse	−0.265 (−0.824, 0.294)	0.348	0.035 (−0.562, 0.632)	0.908
Emotional neglect	−0.218 (−0.476, 0.040)	0.096	−0.209 (−0.619, 0.201)	0.314
Physical neglect	−0.136 (−0.720, 0.448)	0.645	0.210 (−0.416, 0.836)	0.506
Minimization/denial	0.076 (−1.021, 1.173)	0.891	−0.934 (−2.342,0.473)	0.190
**Intimate partner violence**	
**Physical violence**	
No	
Yes	0.082 (−2.324, 2.488)	0.946	0.231 (−2.274, 2.736)	0.855
**Sexual violence**	
No	
Yes	−0.781 (−3.156, 1.593)	0.515	−0.390 (−2.905, 2.124)	0.758

B, regression coefficient; CI, confidence interval.

* *p* < 0.05;

** *p* < 0.01.

## Discussion

This study focused on the history of childhood trauma, IPV, and perceived parenting competency among women in a substance rehabilitation center. This study reports a Malay-predominant sample (92.5%). This is reflective of the substance use population locally, where Malays make up the bulk of PWUD at 82.5% (4). The mean age of the study population is 33.5. Looking at the type of substance used, we found that all these women used ATS before. This is consistent with another study survey conducted in Malaysia looking at the pattern of substance use in Malaysia, with the most commonly used drug across the lifetime being ATS (32). We also discovered that 69.8% of these women came from low-income families, with a monthly income of less than RM 2,000, resulting in a rise in the use of cheaper drugs such as MA tablets compared with other drugs in Malaysia (33). Another possible reason why women use ATS can be explained due to its properties, including elevating mood, boosting energy, and increasing sexual desire, which help them cope with the daily demands and struggles of motherhood. A previous study on MA use among sex workers revealed that amphetamine is a popular option among women because it helps them work longer hours while still increasing their sexual desire, thus enabling them to generate a better income (34).

The study findings reveal that emotional abuse and physical neglect were the two highest childhood traumas experienced, which were reported by 66 and 39.6% of the participants, respectively. A local study conducted among male drug addicts in rehabilitation centers found that emotional abuse and physical abuse were the most reported, which were reported by 50.5 and 38% of the participants, respectively (35). This trend was similar to that reported in other studies on childhood maltreatment, where the most common forms of abuse were emotional abuse and neglect (36, 37). When exploring violence with an intimate partner, 70.5% of these women experienced physical violence from their intimate partner, and 30.5% encountered sexual violence from their partner. A relatively high number of women have undergone at least one form of abuse in their entire relationship with men, either in their previous or current relationship. This finding is similar to those of previous studies reporting that intimate partner abuse is especially high among women with a drug use disorder (15, 38). In another study, IPV was identified in 47–90% of reproductive-age women with a substance use disorder, compared with 1–20% in non-substance use populations (39). It would be possible to say that a majority of these abused women use the substance as a coping mechanism for the trauma they experienced, as discussed by Gezinski et al. (40). Similarly, Hobkirk et al. (41) reported that post-traumatic stress disorder and MA use as a coping strategy were significant mediators for MA addiction among women with a history of IPV.

Previous studies have reported that all childhood traumas are linked with IPV. Particularly, emotional abuse, neglect, and childhood sexual trauma were more emphasized to be important contributors to being victimized by intimate partners as adults, regardless of sexual or physical abuse (37, 42, 43). This study similarly found that women who were sexually victimized by their partners had a higher score in childhood emotional abuse compared with the other domains of childhood trauma than the women who have not experienced sexual violence. This can be understood that emotional abuse in childhood can increase insecure attachment and thus further affect the development of interpersonal and emotional control, making victims more susceptible to re-victimization (42). Similarly, like previous research, it is not surprising that this study found that emotional abuse during childhood is the most common type of abuse that is positively correlated with the occurrence of sexual violence.

Women with children were also assessed for their perception of their parenting abilities. Previous research suggested that parents with a history of trauma have been found to hold more negative views about themselves than their perceived capability to parent a child (36, 44, 45). The findings of this study similarly revealed that parents with a history of childhood maltreatment were negatively correlated with perceived parental competency. Unlike other studies that showed the significance of childhood sexual abuse with lower perceived parenting competence (36, 46), only emotional neglect and emotional abuse and physical abuse were reported as significant in this study. Nonetheless, the findings of this study are line with the study by Baiverlin et al. (45) and Barrett (47), where no difference was found in perceived competence among mothers with and without childhood sexual abuse.

Regarding the components of childhood trauma and parenting competence, there was a significant negative correlation between childhood emotional abuse and physical abuse (non-sexual) with parenting sense of satisfaction. This finding was consistent with that of other research reporting that childhood physical and emotional abuse, neglect, observing domestic violence in childhood, or living apart from one or both parents during childhood was associated with parenting stress (36). Mothers who were sexually abused in childhood provided less positive structure and lower satisfaction (48). A study found that sexually abused mothers were more likely to have psychiatric comorbidities, such as depression and substance use disorder (49), which may play a role in the development of their self-esteem and thus lead to a negative perception of themselves.

Another factor that could contribute to their lack of perceived competence is that parents with a history of childhood trauma are more likely to experience stressors related to lower socioeconomic status (50). These socioeconomic gaps would directly affect parenting capacity and the ability to provide basic needs of children, rather than “parental skills deficiency” within parents (51). However, this was not shown in this study, where no significant correlation between the mothers' socioeconomic status and their perception of parental competency was found. On top of that, experiencing IPV is also an indicator of lower self-competency among adolescent mothers (52). In this study, many respondents answered that having low self-perception of being a good mother, failing at accomplishing in life, being unsure if they were doing an excellent job of parenting, and feeling worthless as a mother may indicate a lower perceived self-competency. This could be largely affected by similar factors explained earlier, such as substance use disorder, other psychiatric comorbidities, and the history of childhood trauma faced by these women (21, 36, 45).

ATS use should not be overlooked when accounting for violent perpetration and victimization. There is a significant difference between the prevalence of IPV among women with ATS use disorder compared with the national average. In this study, about 70 and 30% of these women, compared with the national average of 5 and 1.7%, had experienced physical and sexual violence, respectively (53). A recent study has noted that the prevalence of IPV in Malaysia widely varies, ranging between 4.9 and 35.9% (13). This was further reported in a longitudinal study by Foulds et al. (54), which claimed that MA use is an independent risk factor that raises the risk of violence involved in the general population (54). A similar trend can be seen in perceived parental competence, where MA use may play a role in how these women view themselves as parents. Parents involved with MA showed higher stress and perceived depression in their role as parents. This, coupled with a perception of highly demanding children, leads to the perception of a lack of competency in raising their children (55).

This study has several limitations. First, the sample size of this study was relatively small. It would also be better if the participants included women using ATS out-of-treatment centers. The cross-sectional nature of the data does not allow inferences regarding the causation of the relationship between childhood trauma, IPV, and parenting competency among the population of the study. The study results also relied on retrospective data of childhood trauma and IPV, which may be subject to recall and reporting bias. These data can also be confounded by many factors such as underreporting based on concerns of stigmatization, and the duration, amount, and frequency of drug use, which were not addressed in this study. Many other mediating factors may contribute to re-victimization such as emotional dysregulation, coping skills, and reduced sexual refusal assertiveness, which were not investigated by this study (56).

Furthermore, this study used self-reported data. This may be inaccurate as such data are often underreported. This study also did not explore other psychiatric comorbidities that may have contributed to the occurrence of IPV/substance use such as depression, personality disorders, and attention deficit hyperactive disorder. Further studies should also examine the difference between IPV and mental health problems between ATS-only users and ATS polydrug users. Another limitation of this study is that women were only viewed from the angle as victims and not as perpetrators of IPV, which may be important as ATS use increases the risk of aggression.

Furthermore, the study participants were predominantly from a single ethnic group. Thus, differences in religion, culture, language, and nationality could not be ascertained. This is because the majority of the rehabilitation center inmates were of Malay ethnicity, which is also the leading ethnic group in Malaysia involved with drugs.

Both childhood abuse and IPV play an important role in the perceived parenting competency of women. In addition, these two are also linked to intergenerational transmission of violence. Therefore, it is of great importance for individuals to recognize the risk factors that may predispose them or their loved ones to any form of abuse. We would also like to implore rehabilitation centers for women to include parenting, social, and resiliency skills lessons. These lessons should also include behavior management, juridical education on abuse, and steps for applying for victim assistance (57). To reduce current gaps in responses to violence against women, it is recommended to establish guidelines on responding to domestic violence and child abuse at the primary healthcare level. This is to facilitate primary healthcare providers in recognizing clinical evidence of abuse and equip them with skills to handle such cases. This is especially true given that the prevalence of IPV against women attending primary care clinics was 22% (58).

## Conclusion

Violence against women in the family and society is a key obstacle to the development of society. Given the large number of women with a history of childhood trauma and IPV, recognizing the patterns of violence and parenting competency is crucial to understand the indirect effect of trauma across multiple generations. This study also highlights the importance of non-sexual childhood trauma for being recognized as a predictor of re-victimization.

As trauma and abuse can be intergenerational, we recommend exploring the role of women as perpetrators of IPV and child abuse and how their use of drugs could potentially exacerbate violence. Other than that, it is equally important to investigate the protective roles that women can play to break the cycle of violence.

## Data availability statement

The datasets presented in this article are not readily available because the dataset is protected as per the agreement made with the rehabilitation center. Requests to access the datasets should be directed to suzaily@ppukm.ukm.edu.my.

## Ethics statement

The studies involving human participants were reviewed and approved by the Universiti Kebangsaan Malaysia Ethics Committee and the National Medical Research Register. The patients/participants provided their written informed consent to participate in this study.

## Author contributions

SW: conceptualization, project administration, supervision, funding acquisition, and writing—review and editing. RS: data curation, formal analysis, investigation, methodology, and writing—original draft. AA: formal analysis, methodology, and writing—review and editing. NC: supervision and writing—review and editing. RM: supervision and writing—review and editing. All authors contributed to the article and approved the submitted version.
